# Morning Cortisol and Circulating Inflammatory Cytokine Levels: A Mendelian Randomisation Study

**DOI:** 10.3390/genes13010116

**Published:** 2022-01-08

**Authors:** Skanda Rajasundaram, Rezbieara P. Rahman, Benjamin Woolf, Sizheng Steven Zhao, Dipender Gill

**Affiliations:** 1 Kellogg College, University of Oxford, Oxford OX2 6PN, UK; 2 Faculty of Medicine, Imperial College London, London SW7 2AZ, UK; 3 Clinical Pharmacology and Therapeutics Section, Institute for Infection and Immunity, St George’s, University of London, London SW7 0RE, UK; m1903840@sgul.ac.uk (R.P.R.); dipender.gill@imperial.ac.uk (D.G.); 4 Medical Research Council (MRC) Integrative Epidemiology Unit, University of Bristol, Bristol BS8 1TU, UK; benjamin.woolf@bristol.ac.uk; 5 School of Psychological Sciences, University of Bristol, Bristol BS8 1TU, UK; 6 Centre for Epidemiology Versus Arthritis, Division of Musculoskeletal and Dermatological Science, School of Biological Sciences, Faculty of Biological Medicine and Health, The University of Manchester, Manchester Academic Health Science Centre, Manchester M13 9PG, UK; sizheng.zhao@manchester.ac.uk; 7 Clinical Pharmacology Group, Pharmacy and Medicines Directorate, St George’s University Hospitals NHS Foundation Trust, London SW17 0QT, UK; 8 Department of Epidemiology and Biostatistics, Imperial College London, London W2 1PG, UK; 9 Novo Nordisk Research Centre Oxford, Old Road Campus, Oxford OX3 7FZ, UK

**Keywords:** morning cortisol, steroid, inflammation, cytokines, Mendelian randomisation

## Abstract

Cortisol exerts a broad anti-inflammatory effect on the immune system. Inflammatory cytokines contribute to the molecular signalling pathways implicated in various autoimmune and inflammatory conditions. However, the mechanisms by which cortisol modulates such signalling pathways remain uncertain. Leveraging summary-level data from the CORtisol NETwork (CORNET, *n* = 25,314) and FINRISK (*n* = 8293) genome-wide association studies, we used two-sample Mendelian randomisation to investigate the causal effect of genetically proxied morning cortisol levels on 42 circulating cytokines. We found that increased genetically proxied morning cortisol levels were associated with reduced levels of IL-8 and increased levels of MIF. These results provide mechanistic insight into the immunomodulatory effects of endogenous cortisol and the therapeutic effects of exogenous corticosteroids. Clinically, our findings underline the therapeutic importance of steroids in inflammatory conditions where IL-8 and MIF play a central pathophysiological role in the onset and progression of disease.

## 1. Introduction

Cortisol is an essential steroid hormone released from the adrenal gland. Cortisol levels follow a circadian rhythm under the control of the Hypothalamic–Pituitary–Adrenal axis, reaching their highest levels in the morning. Cortisol has long been known to exert immunosuppressive effects [1] and accordingly, glucocorticoids are the mainstay of clinical management for many autoimmune and inflammatory conditions. However, the molecular mechanisms by which glucocorticoids exert therapeutic effects in inflammatory disease is poorly understood. A role for cortisol in downregulating certain pro-inflammatory cytokines and upregulating other anti-inflammatory cytokines is widely established in the basic science literature [2,3,4], yet there remain gaps in our understanding of precisely how circulating cortisol modulates various cytokines. Studies have historically focused on a small number of well-established cytokines but the effect of cortisol on many important albeit less well-studied cytokines remains uncertain [5]. By understanding how different cytokines are influenced by glucocorticoids, we can better understand the therapeutic mechanisms via which glucocorticoids provide clinical benefit in inflammatory disease. Moreover, by triangulating this information with a growing appreciation of the role of different cytokines in different inflammatory diseases, we may be able to identify novel, more precise immunotherapy targets capable of replicating the therapeutic effects of glucocorticoids whilst avoiding some of their adverse effects. 

Mendelian randomisation (MR) utilises genetic variants to make inferences about the causal effect of an exposure on an outcome [6]. By virtue of Mendel’s Law of Segregation and Law of Independent Assortment, the inheritance of genetic variants is random. This reduces the likelihood that the phenotypic effect of a particular genetic variant is related to, and thus confounded by, environmental factors. Furthermore, germline genetic variation is non-modifiable by the environment and temporally precedes the onset of clinical outcomes, which in turn reduces the risk of reverse causality. Thus, under specific assumptions, Mendelian randomisation allows us to investigate the causal effect of cortisol on a comprehensive range of inflammatory cytokines in a manner that is less vulnerable to certain fundamental weaknesses of traditional observational studies [7,8].

In this study, we performed MR to investigate the effect of genetically proxied morning circulating cortisol on levels of 42 cytokines, chemokines, and growth factors (Table 1).

## 2. Materials and Methods

### 2.1. Overview

**Figure 1:**
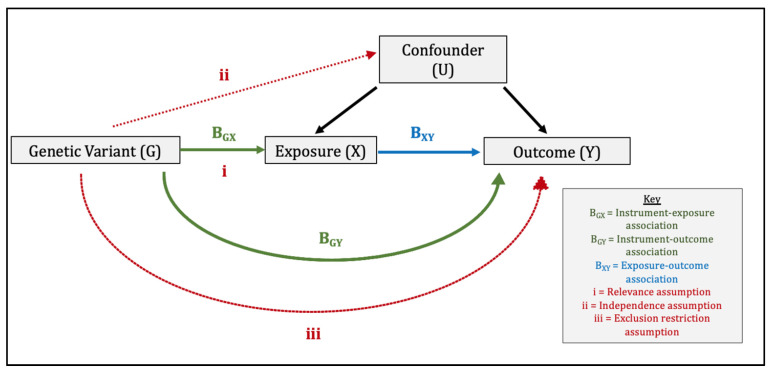
Direct Acyclic Graph Illustrating the Three Core Instrumental Variable Assumptions and Wald Ratio Method used in Mendelian randomisation. The three core instrumental variable (IV) assumptions are: (1) relevance: the genetic variants are associated with the exposure, (2) independence: the genetic variants are independent of confounders, (3) exclusion-restriction: the genetic variants influence the outcome only via the exposure (or factors downstream of the exposure). In the Wald ratio method, the instrument–outcome association (B_GY_) is divided by the instrument–exposure association (B_GX_) to produce a ratio estimate for each genetic variant. These ratio estimates are then combined in an inverse-variance weighted meta-analysis to produce a causal MR estimate of the effect of X on Y.

Mendelian randomisation comprises the use of genetic variants within an instrumental variable (IV) analysis (Figure 1) [6]. For the genetic instrument to be valid, three assumptions must be met: (1) relevance: the genetic variants are associated with the exposure, (2) independence: the genetic variants are independent of confounders, and (3) exclusion-restriction: the genetic variants influence the outcome only via the exposure [6,7]. We performed a two-sample MR study whereby ‘two-sample’ refers to the fact that gene-exposure and gene-outcome estimates are obtained using summary data from two separate genome-wide association studies (GWAS). Consequently, two-sample MR makes two additional assumptions. The first is that the gene-exposure and gene-outcome associations estimated in both samples are representative of the same population, i.e., the same ethnicity, a similar age and sex distribution etc. The second is that of no sample overlap. In our study, there was no overlap between the datasets contributing toward the gene-exposure association and gene-outcome associations, which could otherwise introduce sampling bias [11]. We reported our findings in line with the MR-STROBE guidance [12].

### 2.2. Data Sources

The genetic instrument for morning circulating cortisol was obtained from the Crawford et al. 2021 publication of the CORtisol NETwork (CORNET) genome-wide association study [13] (Table S1). The CORNET GWAS consists of 17 population-based cohorts of European ancestries and 25,314 individuals, with adjustments made for age, sex, and 10 principal components of genetic ancestry in order to reduce the risk of confounding by population stratification. The setting and participants have been described in detail previously in the Supplementary Materials of Crawford et al., where a table of characteristics of the study participants and circulating cortisol measurements can also be found [13]. Briefly, circulating cortisol levels were sampled between 07:00 a.m. and 11:00 a.m. and were quantified via immunoassays of blood samples in all but one cohort, which used liquid chromatography mass spectrometry. Mean circulating cortisol levels across these 17 cohorts ranged from 292 to 979 nmol/L. Some cohorts measured circulating cortisol levels from plasma and others from serum. It should be noted that the ‘morning’ in morning cortisol refers to the measurement timing and is a means of standardising cortisol measurements, as opposed to representing genetically proxied variation in morning levels of circulating cortisol. The instrument consists of four uncorrelated single-nucleotide polymorphisms (SNPs) within 1000 kB of the *SERPINA6/A1* locus on chromosome 14 that reached a genome-wide level of significance (*p* < 5 × 10^−8^) in the CORNET GWAS. SNPs were selected after pruning for linkage-disequilibrium (LD) at *r*^2^ < 0.3 using the TwoSampleMR R package (version 4.1.0). The reference panel was the European panel of 1000 Genomes data (phase 1, release 3). Clumping was performed using the TwoSampleMR R package (version 4.1.0) and the reference panel was the European panel of 1000 Genomes data (phase 1, release 3) [14]. This process ensures that each of the constituent SNPs in the genetic instrument represent independent biological signals, thereby avoiding overestimation of instrument strength and overly precise effect estimates. The *SERPINA1/A6* locus influences the function of cortisol-binding globulin (CBG), a protein that carries cortisol in the plasma [15]. Our genetic instrument collectively explained ~1% (*R*^2^ = 0.954) of the variance in morning cortisol and we calculated a mean F-statistic of 53.9, consistent with a strong relationship between our genetic instrument and exposure phenotype (Table S1).

We sought to investigate the relationship between cortisol and as comprehensive a range of inflammatory cytokines as is feasible, given the existing availability of summary-level genetic data needed for two-sample MR. Summary statistics for the outcome data for 41 of the 42 cytokines were obtained from the Kalaoja et al. 2017 GWAS including 8293 Finnish ancestry individuals from three independent cohorts: the Cardiovascular Risk in Young Finns Study (YFS), FINRISK1997, and FINRISK2002 (Tables S2–S42) [9]. This GWAS represents the largest and most comprehensive meta-analysis of summary-level genetic data for inflammatory cytokines available to date. Adjustments were made for 10 principal components of genetic ancestry, age, and sex in order to reduce the risk of confounding by population stratification. The setting and participant characteristics of these studies are described in detail in an earlier GWAS from Ahola-Olli et al. 2017 [16]. Cytokine quantification was performed from EDTA plasma in FINRISK1997, from heparin plasma in FINRISK2002, and from serum in YFS. The summary statistics for CRP were obtained from the Neale Lab UK Biobank GWAS, in which adjustments were made for 20 principal components of genetic ancestry, age, age^2^, age*sex, and age^2^*sex (Table S43) [10]. Standardised units were used to help deal with non-differential measurement errors arising from different laboratory techniques used to measure circulating morning cortisol and cytokine levels within the exposure and outcome GWAS datasets.

### 2.3. Statistical Analysis

Genetic associations were harmonised by aligning effect alleles in both exposure and outcome datasets, with no exclusions made for palindromic variants. Causal estimates for each variant were generated using the Wald ratio, i.e., the SNP-outcome association divided by the SNP-exposure association [17,18]. The corresponding standard errors for each Wald estimate were approximated using first-order terms from the delta expansion method [19]. For each cytokine, Wald estimates across all four genetic variants were then pooled using the (multiplicative) random-effects inverse-variance weighted (IVW) method [18,19]. The IVW estimate is a weighted mean of the variant-specific estimates with each variant weighted in inverse proportion to its variance and where first-order estimates of the variance of the SNP-outcome association are used. The MR estimates derived represent the association of a 1 standard deviation (SD) increase in genetically proxied morning cortisol levels with each of the respective 42 circulating cytokines, which are themselves normalised to SD units. To account for multiple testing and reduce the risk of false positive findings, a Bonferroni correction was applied (42 significant tests, *p* < 0.00119).

In violation of the exclusion-restriction assumption, horizontal pleiotropy may introduce bias where genetic instruments act on the outcome other than via the exposure (see Figure 1). Consistent estimates from methods with different assumptions about pleiotropy suggest that bias from pleiotropic effects is less likely. The standard IVW method assumes that the average pleiotropic effect is 0, either because all of the variants used are valid instrumental variables or because pleiotropy is balanced [19]. Accordingly, in our secondary analyses, we relaxed these assumptions by using methods that are robust to the inclusion of pleiotropic variants, thereby allowing us to interrogate the risk of bias due to horizontal pleiotropy. Simple median [20], weighted median [20], MR-Egger [21], and MR-PRESSO [22] sensitivity analyses were performed. The MR-Egger intercept estimates the average pleiotropic effect and also provides a significance test for pleiotropy. MR-Egger is based on the INstrument Strength Independent of the Direct Effects (INSIDE) assumption; namely, the magnitude of the pleiotropic effects of the variants on the outcome is independent of the association between the variants and the exposure [21]. MR-PRESSO also includes a global significance test for detecting horizontal pleiotropy. Finally, we used Cochran’s Q statistic to assess whether the heterogeneity between variant-specific causal estimates in our multiplicative random-effects IVW model was more than that expected due to chance alone. Here, significant heterogeneity may indicate the presence of outliers and in turn, pleiotropy [23]. Data analyses were conducted using the “TwoSample MR” and “MendelianRandomization” packages in R (version 4.1.0).

## 3. Results

**Figure 2:**
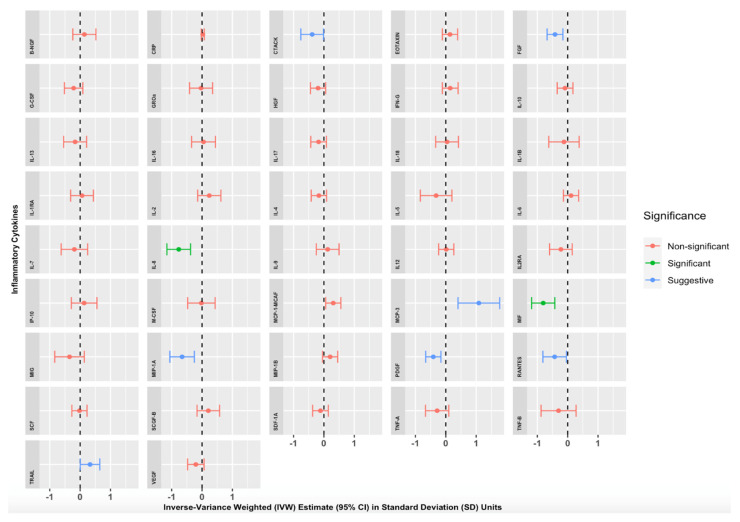
Mendelian randomisation Inverse Variance Weighted (IVW) Estimates for the effect of a 1 Standard Deviation (SD) increase in genetically proxied increased morning cortisol on genetically proxied levels of 42 circulating cytokines. Forest plots showing IVW estimates and corresponding 95% Confidence Intervals for each of the 42 circulating cytokines, expressed in normalised SD units. Green = significant result after Bonferroni correction, *p* < 0.00119). Blue = suggestive result (significant at *p* < 0.05 but non-significant after Bonferroni correction). Red = non-significant result.

We found strong evidence of an association between genetically proxied increased morning cortisol levels and circulating levels of two inflammatory cytokines (Table S44) and Figure 2). A 1 SD increase in genetically proxied morning cortisol levels corresponded to a 0.767 normalised SD-unit decrease in Interleukin 8 (IL-8) (*p* = 1.14 × 10^−4^, 95% CI = −1.157 to −0.378) and a 0.806 normalised SD-unit decrease in Macrophage Migratory Inhibitory Factor (MIF) (*p* = 3.68 × 10^−5^, 95% CI = −1.189 to −0.423). These findings were significant after applying the Bonferroni correction for multiple testing (*p* < 0.00119). Numerous other cortisol-cytokine effect estimates were suggestive of an MR association (with *p* < 0.05) but did not withstand Bonferroni correction: Cutaneous T-cell-attracting Chemokine (CTACK), Fibroblast Growth Factor 2 (FGF2), Monocyte Chemotactic Protein-3 (MCP-3), Monocyte Chemoattractant Protein-1 (MCP-1/MCAF), Macrophage Inflammatory Protein-1 alpha (MIP-1A), Platelet-Derived Growth Factor (PDGF-BB), CC Chemokine Ligand 5 (RANTES), and Tumour-Necrosis Factor-related Apoptosis-inducing Ligand (TRAIL). Estimates were similar with Simple Median, Weighted Median, MR-Egger, and MR-PRESSO sensitivity analyses, suggesting that our results were not biased by pleiotropic associations of the genetic variants. The full results of our primary analyses are summarised in Table S44 and illustrated in Figure 2, where IVW effect estimates and corresponding 95% CIs are given for each cytokine in SD units per 1 SD increase in morning cortisol levels.

## 4. Discussion

### 4.1. Principal Findings in Context

This study provides genetic evidence supporting a causal effect of increased morning cortisol levels on reducing circulating IL-8 and MIF levels.

Both IL-8 and MIF are key components of the innate immune system. Classically, IL-8 is a chemokine released by macrophages via toll-like receptor (TLR) pathways that promote the recruitment of neutrophils and other granulocytes to new sites of inflammation via chemotaxis [24]. Upon the arrival of neutrophils, IL-8 also induces phagocytosis by neutrophils [24]. In contrast to IL-8 secretion—and indeed the secretion of most other cytokines—MIF is constitutively expressed by macrophages, T cells, and nearby epithelial cells [25]. Elevated baseline expression of MIF by macrophages translates into upregulated TLR signalling and ultimately facilitates the rapid detection of endotoxin-containing bacteria by macrophages [26,27]. MIF also acts as a traditional pro-inflammatory cytokine by activating macrophages and T cells and by engaging in bidirectional feedback loops with various other pro-inflammatory cytokines [25]. Hence, MIF-deficient mice fail to produce an adequate pro-inflammatory cytokine response to intracellular pathogens and are simultaneously unable to control the growth of such pathogens in comparison to wild-type (WT) mice [28]. Thus, the uniquely constitutive expression of MIF in addition to its traditional pro-inflammatory signalling properties make it integral to the innate immune response to invasive pathogens. 

A range of evidence corroborates our findings. Our finding that cortisol exerts an inhibitory effect on IL-8 is supported by molecular studies in a range of cell lines, which have shown that steroids inhibit IL-8 gene transcription via glucocorticoid response elements in the IL-8 gene [29]. By leveraging genetic data at a large scale, our study provides an important additional source of evidence for the inhibitory effect of steroids on IL-8. Furthermore, our finding that higher genetically proxied morning cortisol levels are associated with reduced circulating MIF is consistent with the overall immunosuppressive effect of glucocorticoids in two distinct ways. Firstly, considering the protective role of MIF in responding to pathogenic infections, our finding is consistent with the increased susceptibility to pathogenic infections seen with corticosteroids in clinical practice. Secondly, considering MIF’s classical pro-inflammatory signalling functions, our finding is consistent with the marked anti-inflammatory effects of glucocorticoids. 

Interestingly, however, the inhibitory effect of steroids on MIF observed in our study runs counter to the established finding that exogenous glucocorticoids *induce* the release of MIF, in contrast to their uniform suppression of other pro-inflammatory cytokines [30]. This discrepancy likely reflects the biphasic and bidirectional nature of the interaction between glucocorticoids and MIF in the regulation of innate immunity. Indeed, whilst glucocorticoids have been shown to induce MIF, MIF has consistently been shown to inhibit and counter-regulate the action of glucocorticoids [30]. Furthermore, it has been shown that the ability of exogenous glucocorticoids to induce MIF release is greatest at low physiological concentrations and diminishes at increasing concentrations [30,31,32]. In other words, the stimulatory effect of glucocorticoids on MIF may not hold in circumstances in which glucocorticoid concentrations are significantly elevated, beyond this low physiological range. Indeed, it is likely that the effect of gradual changes in glucocorticoid concentrations studied using in vitro and in vivo cell lines will differ to that in MR, which estimates the lifelong effect of expressing additional glucocorticoid level-increasing alleles and thus represents the effect of marked upward shifts in glucocorticoid concentrations. Taken together, these findings suggest a complex, concentration-dependent feedback loop between glucocorticoids and MIF that will require additional studies using a range of molecular and epidemiological techniques in order to better elucidate.

### 4.2. Clinical Implications

Corticosteroids are widely used in the clinical management of inflammatory diseases and our study offers insight into the mechanisms by which steroids exert their therapeutic effect. Specifically, our results suggest that the therapeutic effect of steroids is in part mediated by their inhibition of the pro-inflammatory effects of IL-8 and MIF. This in turn highlights the therapeutic value of steroids in conditions where IL-8 and MIF play a central role in disease onset and progression. Of note, both IL-8 and MIF have consistently been implicated in propagating inflammatory cascades in Inflammatory Bowel Disease (IBD) [33,34,35,36,37,38,39,40], Rheumatoid Arthritis (RA) [41,42,43,44,45,46], Systemic Lupus Erythematosus (SLE) [47,48,49].

With respect to IBD, IL-8 gene expression is increased in the bowel mucosa and epithelial cells of patients with Crohn’s and Ulcerative Colitis [33,34] and is associated with the histological grade of inflammation [35]. IL-8 has even been shown to predict the risk of relapse in Crohn’s patients in remission [36]. Certain polymorphisms in the MIF gene are also associated with an increased risk of developing IBD in patients [37]. Conversely, administering anti-MIF antibodies has been shown to reduce the degree of inflammation in experimental models of inflammatory colitis and MIF-deficient mice are resistant to developing inflammatory colitis in the first place [38]. Similarly, numerous studies demonstrate a role for IL-8 [39,40] and MIF [41,42] in propagating synovial inflammation in Rheumatoid Arthritis and MIF gene promoter polymorphisms have been shown to correlate with the 28-joint Disease Activity Score [43]. Patients with SLE tend to have higher circulating IL-8 levels and this correlates with the SLE disease activity index [44,45]. IL-8 gene polymorphisms are even associated with the severity of Lupus Nephritis [46]. In SLE, MIF exerts a paradoxical effect that reflects its dual immune functions [47]. Circulating levels of MIF are increased in SLE patients and are associated with autoimmune tissue damage, including lupus nephritis and subsequent renal dysfunction [48]. However, high expression MIF alleles reduce the risk of less secondary pathogenic infections in patients with SLE [49], consistent with its important role in the immunological alarm system. 

Taken together, these findings suggest that the strong efficacy of corticosteroids in treating these specific conditions may in part be attributable to the ability of steroids to regulate IL-8 and MIF activity. It also follows that IL-8 and MIF could represent alternative therapeutic targets in IBD, RA, and SLE which, if precisely targeted, may avoid some of the adverse effects associated with long-term corticosteroid therapy [50]. Alternatively, specifically targeting IL-8 or MIF could offer additional treatment options to patients with IBD, RA, and SLE who for various reasons do not respond to steroids.

### 4.3. Strengths

Our study has many strengths. Firstly, it is the first MR analysis to investigate the causal effect of morning cortisol on a comprehensive panel of inflammatory cytokines. Many of these cytokines have well-established roles in autoimmune and inflammatory diseases whilst others are poorly understood, especially in the context of cortisol-mediated immune regulation. Recent studies have adopted a similar approach to investigating the role of inflammatory cytokines in immune signalling pathways [5]. Secondly, we leveraged genetic data in the MR framework and thus were able to produce evidence of causal associations less vulnerable to confounding and reverse causality in comparison to conventional observational studies. Thirdly, in order to minimise pleiotropy, genetic variants were chosen based upon their proximity to the *SERPINA1/A6* genes, which is implicated in mediating the physiological effects of plasma cortisol. Expression quantitative trait loci analyses have shown that the *SERPINA1/A6* locus contains genes encoding cortisol-binding globulin (CBG) and may therefore facilitate cortisol delivery to peripheral tissues [12]. Thus, in addition to a very strong statistical association (*p* < 5 × 10^−8^), the presence of an established biological link between our selected variants and exposure of interest strengthens the validity of these variants as instruments within the IV framework. This strengthens our conclusion that the MR estimates generated in this study reflect causal relationships rather than mere association. Fourthly, we interrogated the robustness of our IVW estimates to the presence of pleiotropic variants using a range of sensitivity analyses, each of which make different assumptions, and we did not find evidence of significant pleiotropy. Rather, our MR estimates were consistent across these different robust methods. Finally, there was no significant heterogeneity between variant-specific estimates, consistent with a concordant causal effect across multiple genetic variants.

### 4.4. Limitations

Our analysis has a number of limitations, primarily with regard to its scope, which should inform the interpretation of our results. Given that genetic variants are present in the germline, MR estimates represent the effect of lifelong elevated levels of morning cortisol starting from conception, rather than the effect of a discrete clinical intervention that increases morning cortisol levels in mature individuals, in whom a degree of disease progression is already manifest [8]. Nor can the effect of lifelong elevated morning cortisol levels be extrapolated to reflect the impact of acute changes in morning cortisol levels on circulating cytokines. Our estimates should not be extrapolated to estimate the effect of changing morning cortisol levels in subgroups of the population with particularly high or low morning cortisol levels [21]. The pooling of genetic data across numerous cohorts in consortia-derived genome-wide association studies will give rise to a degree of heterogeneity between cohorts and yet the combined cohort is treated as a homogenous population in our MR analysis. Measurements differed across the individual cohorts in the underlying GWAS datasets in terms of both laboratory techniques used to measure cortisol and cytokine levels, as well as protocols for sampling plasma or serum levels of cortisol and cytokines. Although standardised units were used to help account for these differences, there will nevertheless be a degree of measurement variability in both our exposure and outcome. However, given the large sample size in this study, such variability is unlikely to significantly affect the magnitude or directionality of our MR estimates. Unfortunately, because we only had access to summary-level and not individual participant-level genetic data, we are not able to perform sub-group analysis investigating whether the relationship between cortisol and particular cytokines varies between males and females or across different age groups. The summary-level genetic data used for both exposure and outcome were obtained from individuals of European ancestry. Given that the frequency and distribution of genetic variants may differ across populations, the extent to which these findings can be generalised to other ethnic populations remains uncertain [7,8]. Finally, despite finding no evidence of significant pleiotropy in our sensitivity analyses, we cannot definitively exclude the potential for bias due to horizontal pleiotropy whereby the variants used to proxy morning cortisol levels influence circulating cytokine levels via pathways independent of morning cortisol levels.

## 5. Conclusions

In summary, this study identified a novel causal effect of increased genetically proxied morning cortisol on circulating levels of IL-8 and MIF. Such findings provide useful mechanistic insight into the immunomodulatory effects of endogenous cortisol and the therapeutic effects of exogenous glucocorticoid therapy. This bears clinical relevance to inflammatory diseases where IL-8 and MIF play a central pathophysiological role in the initiation and progression of disease.

## Figures and Tables

**Table 1:** The 42 cytokines, chemokines and growth factors upon which the effect of morning cortisol was investigated.

Cytokine or Growth Factor
Beta-nerve growth factor (B-NGF)
Cutaneous T-cell-attracting chemokine (CTACK)
Eotaxin
Fibroblast growth factor 2 (FGF2)
Granulocyte-colony stimulating factor (G-CSF)
Growth regulated oncogene-alpha (GROa)
Hepatocyte growth factor (HGF)
Interferon gamma (IFN-G)
Interleukin 1 beta (IL-1B)
Interleukin 1 receptor alpha (IL-1RA)
Interleukin-2 (IL-2)
Interleukin-2 receptor alpha (IL-2RA)
Interleukin-4 (IL-4)
Interleukin-5 (IL-5)
Interleukin-6 (IL-6)
Interleukin-7 (IL-7)
Interleukin- 8 (IL-8)
Interleukin-9 (IL-9)
Interleukin-10 (IL-10)
Interleukin-12-P70 (IL-12-P70)
Interleukin-13 (IL-13)
Interleukin-16 (IL-16)
Interleukin-17 (IL-17)
Interleukin-18 (IL-18)
Interferon gamma-induced protein (IP-10)
Macrophage colony-stimulating factor(M-CSF)
Monocyte chemoattractant protein-1/Monocyte chemotactic and activating factor (MCP-1/MCAF)
Monocyte chemotactic protein-3 (MCP-3)
Macrophage migration inhibitory factor (MIF)
Mitogen-inducible-gene (MIG)
Macrophage inflammatory protein- 1 alpha (MIP-1A)
Macrophage inflammatory protein-1 beta (MIP-1B)
Platelet-derived growth factor (PDGF-BB)
Chemokine ligand 5 (RANTES)
Stem cell factor (SCF)
Stem cell growth factor- beta (SCGF-B)
Stromal cell-derived factor-1 alpha (SDF-1A)
Tumour necrosis factor-alpha (TNF-A)
Tumour necrosis factor-beta (TNF-B)
Tumour necrosis factor-related apoptosis-inducing ligand (TRAIL)
Vascular endothelial growth factor (VEGF)
C-reactive protein (CRP)

The 42 cytokines investigated in this study. Summary statistics for 41 cytokines were obtained from the Kalaoja et al. 2017 GWAS [9]. Summary statistics for CRP were identified from the Neale Lab UK Biobank GWAS [10]. Table adapted from Rahman et al. 2021 [5].

## Data Availability

All data used in this study are publicly available, with corresponding citations detailed. The R source code can be found at https://cran.r-project.org/web/packages/MendelianRandomization/index.html.

## Supplementary Materials

The following are available online at https://www.mdpi.com/article/10.3390/genes13010116/s1, Tables S1–S43 summarise the genetic associations of the four SNPs used in the genetic instrument for morning cortisol with our exposure and outcomes of interest. Table S44: Mendelian randomisation estimates for the effect of a 1 Standard Deviation (SD) increase in genetically proxied increased morning cortisol on genetically proxied levels of 42 circulating cytokines.
